# BMI and Physical Activity Among at-Risk Sixth- and Ninth-Grade Students, Hillsborough County, Florida, 2005-2006

**Published:** 2010-04-15

**Authors:** Heather Agazzi, Kathleen Armstrong, Kathy L. Bradley-Klug

**Affiliations:** University of South Florida, 13101 N Bruce B. Downs Blvd, Tampa, FL; University of South Florida, Tampa, Florida; University of South Florida, Tampa, Florida

## Abstract

**Introduction:**

During the past 3 decades, the number of overweight adolescents increased while adolescent engagement in physical activity decreased. We investigated the prevalence of overweight and physical activity levels among economically disadvantaged and minority middle- and high-school students in a school district in Florida. In particular, data on physical activity levels of middle-school students are limited and needed for prevention and intervention planning. In addition, because of state education policies, students in Florida are less likely than students nationally to enroll in physical education, placing them at a higher risk for decreased physical activity levels.

**Methods:**

We used multivariate methodology to analyze physical activity levels among 526 students from 3 middle and 2 high schools in southwest Florida.

**Results:**

Forty percent of students met criteria for overweight or obesity. Overall, less than 45% of students reported engaging in daily physical activity. Boys reported higher levels of physical activity than did girls, and a decline in physical activity levels was observed between grades 6 and 9, especially among minority girls (ie, African American and Latino). Lack of time was identified as the greatest barrier to engaging in physical activity.

**Conclusion:**

This study documents health disparities among minority students from economically disadvantaged backgrounds in an urban school district. Participation in daily physical activity was below recommended guidelines across grades. We found numerous barriers to engaging in physical activity, which will enable local education agencies to evaluate their current physical activity policies and identify alternative physical activities for these youth.

## Introduction

Physical activity helps to develop and maintain healthy bones, muscles, and joints in youth; to increase flexibility, balance, and endurance for all ages; and to prevent or delay the development of high blood pressure, cardiovascular disease, and diabetes in adults (1). Physical activity boosts psychosocial well-being by increasing self-esteem and self-efficacy and reducing anxiety and depression (2,3). Physical activity also reduces the risk of becoming overweight, which is increasingly recognized as a serious public health issue affecting children and adolescents (4). The proportion of young Americans who are overweight increased from approximately 5% in the early 1970s to 15% in 2000 (5).

The 2005 US dietary guidelines for Americans suggest that children should participate in a minimum of 60 minutes of moderate to vigorous physical activity (VPA) on most (or all) days of the week (6). On the basis of data from the National Health Interview Survey, the Centers for Disease Control and Prevention (CDC) reported that only 65% of adolescents in grades 9 through 12 engaged in the recommended amount of VPA in 1999. As a result, CDC developed a physical activity and fitness objective for adolescents in the *Healthy People 2010* campaign: to increase the proportion of adolescents who engage in moderate physical activity (MPA) or VPA that promotes cardiovascular fitness at least 5 days per week for 30 or more minutes per session (7).

In the state of Florida, participation in daily physical education (PE) classes continues to decline, and Florida high school students are less likely to engage in PE than are US high school students generally (8). Furthermore, Florida high school students can enroll in personal fitness through the Florida Virtual Education classroom, a virtual classroom in which students self-report their eating habits, physical activity, and heart rate (9). Because of growing demands to increase instruction in reading, writing, and mathematics to prepare students for high-stakes testing, virtual PE may also become a popular option in other states.

Physical activity in youth is measured a number of ways, including self-report instruments and more objective measures such as accelerometry and pedometry. Self-report instruments are often used in school settings because they are inexpensive, quick, can be administered by school staff, and are considered appropriate measures for students in grades 7 and higher (10). However, self-report instruments can be challenging for some youth because of their difficulty comprehending concepts of time, duration, and intensity (11), and their desire to respond in a socially acceptable manner (12). Limited data are available on the physical activity of youth in middle school because of a lack of tools designed specifically for this age group (13).

We summarize the results of a collaboration between school district and university staff to determine the prevalence of overweight and physical activity levels among sixth- and ninth-grade students. We developed an instrument to address the aforementioned concerns with self-report instruments for middle-school students and to target the specific variables of interest to the school district personnel, including ease of survey administration.

## Methods

### Setting

Hillsborough County is located on the southwest coast of Florida; approximately 191,000 students are enrolled in its public schools. Forty-one percent of district students are white, followed by Latinos (28%) and African Americans (22%). Approximately 50% of the students were eligible for reduced-price or free lunch in the fall of 2007 (14), a metric often used as an indicator of socioeconomic status. Three middle schools and 2 high schools participated in this study because they were pilot schools for the Steps to a Healthier Hillsborough Initiative Program. This initiative is funded by the US Department of Health and Human Services; its focus is to promote health among county residents by addressing risk factors associated with obesity, overweight, asthma, and diabetes.

### Study sample

We collected data on student demographics, weight, height, age, dietary intake, and physical activity. A total of 535 sixth- and ninth-grade students who were present during PE or health classes on the date of site data collection completed the Nutrition and Exercise Survey for Students (NESS) (15). Health and PE classes were chosen because all sixth- and ninth-grade students in the district are required to take these classes. These schools had a high enrollment of minority students (Latinos and African Americans) (71%-89%), and most students were eligible for free or reduced lunch (51%-82%), which represented an economically disadvantaged sample. Weight and height screenings were completed in November 2005, and physical activity data were collected in March and April 2006. Each participating school completed height and weight screenings and survey administration in 1 academic day, on different dates. Only students who were present on the date of survey administration participated, and no make-up opportunities were provided. Students could refuse participation, but no refusals were reported. Participation rates varied (Table 1) and were particularly low in high schools A and B because only 2 teachers participated. Middle school C had a low participation rate because many students were participating in an off-campus service activity on the day of data collection. Students who did not report grade, sex, age, or race/ethnicity were excluded from analyses, as were those with BMI percentiles <5th for age and sex.

Data were collected in cooperation with school health services, the PE department, and staff from the Steps to a Healthier Hillsborough Initiative Program. The University of South Florida institutional review board approved the instrument and research protocol.

### Measures

We created the NESS because district staff wanted to measure a specific combination of variables (demographics, nutrition, and physical activity) in a manner that was easy for teachers and school nurses to administer and simple for students in sixth and ninth grades to complete. The principal investigator (H.A.) conducted key informant interviews with experts from exercise physiology, nutrition, child development, and educational measurement to develop and review all items on the NESS. We pilot tested the items across sites with sixth- (n = 48) and ninth-grade students (n = 199) in a group format to assess comprehension and face validity, both of which were judged satisfactory. The final survey was 25 items that could be completed in 10 to 15 minutes and assessed demographics (10 items), dietary intake (7 items), and physical activity (8 items). The NESS yielded a Flesch Reading Ease score of 62.9 and a Flesch-Kincaid Grade Level score of 8.1, suggesting that the survey was a standard read at approximately the 8th-grade level. Item-to-total correlations and Cronbach α were obtained for items on the NESS and correlations were moderate (0.64 for sixth-grade students, 0.73 for ninth-grade students).

The school nurse calculated each participant’s age- and sex-specific BMI percentile by using CDC National Center for Health Statistics 2000 growth charts (16). Participants were weighed on beam balance scales and measured without shoes standing erect against a wall-mounted scale. Four weight categories were delineated on the basis of definitions by the Expert Committee on the Assessment, Prevention, and Treatment of Child and Adolescent Overweight and Obesity (11): 1) expected weight for age and sex (BMI percentile ≥5th and <85th), 2) overweight for age and sex (BMI percentile ≥85th and <95th), 3) obese for age and sex (BMI percentile ≥95th), and 4) underweight for age and sex (BMI percentile <5th).

Demographics were based on student self-report to demographic items on the NESS. Although sex, grade, race/ethnicity, and age (for BMI percentile calculations) were the key demographic variables in this study, other variables included eating habits, dieting, and perceived stress. The physical activity items came from the 2003 Youth Risk Behavior Surveillance System (8) and were slightly modified to ensure comprehension and content validity. Physical activity was addressed by items 1 through 3, physical inactivity with item 7, and barriers to physical activity with item 8 (Appendix). Students could select from 8 responses (0-7 days) for questions 1-3, 7 responses (0-5 hours per day) for question 7, 1 or more from 7 responses for question 8, or they could pencil in a unique response for question 8. Cronbach α (questions 1-3) was calculated, and coefficients of 0.76 and 0.77 were obtained for sixth- and ninth-grade students, respectively.

### Statistical analysis

Data were entered into a spreadsheet and analyzed with SPSS version 14.0 for Windows (SPSS, Inc, Chicago, Illinois). Descriptive statistics were calculated for sociodemographic variables and physical activity behaviors. Two multivariate analyses of variance were used to explore differences in physical activity behaviors between groups (ie, weight category, sex, and race/ethnicity) for sixth- and ninth-grade students separately, and missing data were excluded listwise, so that if any value were missing for a participant, the entire record was deleted. The tests were followed by univariate tests for each dependent variable, and effect sizes were reported.

## Results

### Demographics

Most participants (60%) were in the expected weight range (Table 2). Underweight participants (n = 15) and participants with incomplete surveys (n = 9) were excluded from analyses. Approximately 53% of participants were African American, followed by Latinos (34%) and whites (13%); 66.5% were in the sixth grade, and 52.5% were boys.

Students' physical activity levels were compared with CDC's 2005 recommendations for physical activity (8). In general, less than 30% of students across all groups reported engaging in daily physical activity (Table 3). However, students reported engaging in more VPA than MPA. Boys generally reported higher levels of physical activity than girls, and sixth-grade students typically reported more physical activity than ninth-grade students did. For sedentary activity, sixth- and ninth-grade students across all groups (weight category, sex, and race/ethnicity) reported an average of 2 to 3 hours per day.

### Physical activity levels for sixth- and ninth-grade students

For sixth-grade students, there were no interactions between the independent variables weight category, sex, and race/ethnicity. A main effect was observed only for sex (Wilks λ = 0.96, *F* [4, 315] = 2.98, *P* = .02). Tests of between-subjects effects for sex were significant on total activity (*P* = .001) and vigorous activity (*P* = .01). Boys reported a higher mean score on total activity than girls (mean [SD], 4.43 [2.14] and 3.54 [2.26], respectively), although the effect size was small (*d* = 0.40). Boys also reported more vigorous activity than did girls (3.71 [2.18) = 0.40). Boys also reported more vigorous activity than did girls (3.71 [2.18) and 3.06 [2.33], respectively), but the effect size was small (*d* = 0.29).

For ninth-grade participants, there was an interaction between weight category and race/ethnicity (Wilks λ = 0.89, *F* [8, 284] = 2.04, *P* = .04), and tests of between-subjects effects were not significant. No main effects were observed.

### Comparison of physical activity levels between sixth- and ninth-grade students

No significant differences were observed between physical activity levels of sixth- and ninth-grade students (Wilks λ = 0.97, *F *[3, 507] = 2.38, *P *= .07). However, sixth-grade students consistently reported higher levels of physical activity than did ninth-grade students. These data may suggest a slight decline in physical activity levels as students advance through grades.

### Barriers to engagement in physical activity

A total of 312 students responded to the item on barriers to engaging in daily physical activity (sixth-grade students, n = 185; ninth-grade students, n = 127). Students could choose more than 1 response. We analyzed these data descriptively; overall, both grade groups most commonly selected the response "I don't have time" as a barrier to engaging in daily physical activity (Table 4). The second most cited response was "other," and students wrote an answer that best described their personal situation. A review of these "other" responses suggested that most items could have been classified into 1 of the forced-response categories, including "I don't like it," and "I'm not good at it." Some students indicated that the heat in Florida was a factor for not engaging in daily physical activity.

## Discussion

Results of this study, unexpectedly, indicated that the prevalence of overweight and obesity in the sample was greater than the statewide prevalence for Florida high school students (40% compared with 26.4%, respectively) (17). However, our study included a majority (87%) of students from ethnic/racial minority groups. Strauss and Pollock (18) documented that during 1986-1998, overweight increased fastest among Southerners and minorities, specifically African American and Latino populations. Our data support this trend and suggest that students who are minorities, economically disadvantaged, and reside in a Southern state face health disparities.

Overall, approximately 28% of ninth-grade students met the 2005 VPA guidelines, and approximately 23% met the MPA guidelines. These numbers fall short of the *Healthy People 2010* goal of 85% of high school-age students engaging in 5 or more days per week of MPA or VPA for 30 minutes per session (7). Although the sixth-grade students in this sample did not meet recommendations for high school-age students, they reported higher levels of physical activity than ninth-grade students did. Consistent with previous research (19), results demonstrated that physical activity levels decline among youth over time. Girls consistently reported lower levels of physical activity than did boys, and minority girls reported the largest reduction in physical activity between grades 6 and 9. This downward trend of physical activity over time in girls is a health concern that warrants further investigation. Physical activity is important for developing bone density and for prevention of osteoporosis (1), and reducing anxiety and depression (2,3), which are more common among women than men. Consequently, girls should be taught healthy behaviors that protect them against the development of disease.

Future studies should explore factors that prevent youth from participating in physical activity. Not having time to engage in physical activity was reported as the greatest barrier to exercise and may indicate the limited opportunities to engage in physical activity during school hours. All sixth-grade students in this sample attended PE daily throughout the school year; however, the ninth-grade students were required to take only 1 semester of PE and 1 semester of health, which reduced their opportunity for physical activity during the school day. In addition, given the sociodemographic characteristics of this sample, we hypothesize that a lack of time to engage in physical activity may be associated with competing responsibilities such as after-school employment, care of younger siblings, or assistance with other domestic duties. Furthermore, a relatively large portion of sixth- (14.0%) and ninth-grade (16.5%) students reported that they did not like physical activity. Options such as dance, yoga, or virtual exercise video games should be explored to motivate these students and enhance their enjoyment of physical activity.

This study has implications for local education agencies and can be conceptualized within the public health model of primary, secondary, and tertiary prevention. In this approach, intervention and prevention are not mutually exclusive; instead, different approaches are used at each level to achieve specific outcomes (20). At the district level, supervisors of PE and health and student support services (eg, nursing, psychology, social work) could revisit policies regarding PE requirements and opportunities for recess. At the primary prevention level, districts could increase all students' opportunities to engage in physical activity.

On the basis of the results of the present study and the literature on early intervention, sixth-grade students could be targeted for school-wide prevention programs to increase health-supporting activity behaviors. Health professionals could assist in prevention efforts by serving on a school-wide staff committee to develop a healthy behaviors campaign for students to learn about and engage in health-promoting behaviors in the educational setting.

Secondary prevention efforts aim to provide at-risk students with skill development, support, and mentoring (21). Examples of secondary prevention strategies include small-group exercise lessons, behavioral contracting (setting exercise goals and monitoring progress), specialized counseling from a school nurse, and special exercise groups for students at highest risk. Because some youth (eg, girls, minorities) are at higher risk for overweight and insufficient physical activity, secondary prevention strategies should target these subgroups. In addition, because students tend to become less active over time, secondary prevention programs should target younger adolescents who are at risk for decreased activity.

Finally, tertiary prevention is appropriate for students already identified as overweight. Linking these children to community resources that serve youth who are overweight and providing them with mental health counseling to promote their social and emotional well-being also is important.

This study has several strengths. First, trained nursing staff assessed students' BMIs, and the research protocol was carried out exclusively by school staff. Also, the survey was written at an appropriate reading level for most ninth-grade students. Therefore, the participants interacted with people with whom they were familiar and were able to understand the survey. The study took place in schools largely serving minority and economically disadvantaged students and documented the disparity in health factors at young ages.

This study had limitations. For 1 school district, we used a convenience sample of students in grades 6 and 9 in which the majority of students were eligible for free and reduced lunch, and the student body consisted mostly of ethnic and racial minorities. Although these results may not be generalized to all schools, they are important because they provide information relevant to student populations that have been identified as most at-risk for obesity and inadequate physical activity. Future studies should investigate these variables in more representative samples. Second, the readability of the questions may have been challenging for some of the sixth-grade students. Third, data were collected via self-report, a process that has known limitations. Finally, test-retest reliability was not assessed and would be useful in evaluating the reliability of this survey with students in different grades.

In summary, this article describes a collaborative effort between a local university and school district that provided data on a public health issue. Further, it documents the importance of prevention and intervention efforts for sixth- and ninth-grade students to promote better health through increased physical activity.

## Figures and Tables

**Table 1:** Characteristics of Schools and Samples, BMI and Physical Activity Survey, Hillsborough County, Florida, 2005-2006

**School**	Enrollment	Minority, %	Eligible for Free/Reduced Lunch, %	Grade Enrollment	No. of Participants (%)
HS A	2,093	71	51	553	80 (14.5)
HS B	1,707	89	64	484	123 (25.4)
MS A	611	82	74	200	142 (71.0)
MS B	908	83	76	330	219 (66.4)
MS C	834	89	82	275	100 (36.4)

Abbreviations: BMI, body mass index; HS, high school; MS, middle school.

**Table 2:** Sample Characteristics of Participants (N = 535) in BMI and Physical Activity Survey, Hillsborough County, Florida, 2005-2006

**Characteristic**	**Obese, n (%)^a^**	**Overweight, n (%)^a^**	**Expected Weight, n (%)^a^**
**Total**	115 (21.5)	100 (18.7)	320 (59.8)
**Race/ethnicity**			
African American	58 (20.5)	50 (17.7)	175 (61.8)
Latino	44 (24.2)	36 (19.8)	102 (56.0)
White	13 (18.6)	14 (20.0)	43 (61.4)
**Sex**			
Male	59 (21.0)	55 (19.6)	167 (59.4)
Female	56 (22.0)	45 (17.8)	153 (60.2)
**Grade**			
Sixth (n = 362)	83 (23.0)	60 (16.6)	219 (60.4)
Ninth (n = 173)	32 (18.5)	40 (23.1)	101 (58.4)

Abbreviation: BMI, body mass index.

a All percentile scores are age- and sex-specific. Obese, BMI percentile ≥95th; Overweight, BMI percentile ≥85th and <95th. Expected weight, BMI percentile <85th and ≥5th.

**Table 3:** Percentage of Students (N = 526) Who Met Recommended Levels of Physical Activity, BMI and Physical Activity Survey, Hillsborough County, Florida, 2005-2006

**Characteristic**	**Engaged in Daily Physical Activity^a^**		**Met 2005 Recommendations for Vigorous Activity^b^**		**Met 2005 Recommendations for Moderate Activity^c^**	

	6th Grade (n = 356), %	9th Grade (n = 170), %	6th Grade (n = 356), %	9th Grade (n =170), %	6th Grade (n =356), %	9th Grade (n=170), %
**Weight**						
Expected weight	19.2	13.1	59.2	52.0	27.3	20.4
At Risk	33.9	7.7	72.9	61.5	25.9	30.8
Overweight	19.3	12.5	57.0	59.4	30.5	22.6
**Sex**						
Male	27.8	17.2	69.0	65.1	30.8	29.4
Female	14.8	6.0	52.1	45.8	24.6	16.9
**Race/ethnicity**						
African American	25.0	11.8	63.1	55.0	31.9	22.9
Latino	15.9	10.8	56.9	56.8	21.8	16.7
White	27.7	13.0	66.0	56.5	31.9	34.8
**Total sample**	44.6	38.2	32.8	27.8	27.8	23.2

Abbreviation: BMI, body mass index.

a Daily physical activity is at least 20 minutes or longer of any physical activity without stopping.

b Vigorous activity is 20 minutes of vigorous-intensity physical activity on 3 or more days per week.

c Moderate activity is 30 minutes of moderate-intensity physical activity on 5 or more days per week.

**Table 4:** Student-Perceived Barriers to Engaging in Physical Activity by Grade, BMI and Physical Activity Survey, Hillsborough County, Florida, 2005-2006^a,b^

**Barrier**	**Sixth-Grade Students (n = 185), n (%)**	**Ninth-Grade Students (n = 127), n** (%)
I don't like it	26 (14.0)	21 (16.5)
I think it is boring	19 (10.2)	12 (9.4)
Other kids make fun of me	11 (5.9)	7 (5.5)
I'm not good at it	27 (14.5)	9 (7.1)
I don't have time	50 (27.4)	39 (30.7)
It makes me smell bad	11 (5.9)	5 (3.9)
I'm not coordinated	11 (5.9)	9 (7.1)
Other	30 (16.2)	25 (19.7)

Abbreviation: BMI, body mass index.

a Total responses: n = 312.

b Students could choose more than 1 response.

## Appendix

The Nutrition and Exercise Survey for Students is available for download as a Microsoft Word document.

**Download the Nutrition and Exercise Survey for Students** (DOC 152k)

**Description: **Microsoft Word is a word processing program used to create and edit text documents. Text in Word documents can be easily modified or copied for use in other applications.

**File extensions: **.doc, .rtf

**Viewing: **If you do not already have Word, you can download Word Viewer for free.
